# ADVANCE CARE PLANNING AND THE MILITARY EXPERIENCE: FROM ACTIVE DUTY TO VETERAN

**DOI:** 10.1093/geroni/igae098.0944

**Published:** 2024-12-31

**Authors:** Maresi Berry-Stoelzle

**Affiliations:** Iowa City VA Medical Center, Iowa City, Iowa, United States

## Abstract

Active duty military are one of the few groups who are required to face the possibility of mortality and morbidity while young. Pre-deployment paperwork for preparing a servicemembers and their family include the designation of a healthcare power of attorney. Despite this previous experience, rates of advance directive completion (ACP) and documented serious illness conversations (SICC) in VA served Veterans are comparable with the civilian population. Discussing goals and attitudes towards managing current and future healthcare is often sidelined for more immediately urgent medical needs. However, from our recent cohort of Veterans with an inpatient admission, 18% of Veterans admitted inpatient died within a year after their hospitalization, comparable to recent US wide data. Our previous work explores how personalization can help engage Veterans in serious illness conversations. Extending this, we explore how particularly pre-deployment planning organizational structures and discussion formats differ in structure and uptake. We coordinated with local palliative care, social work and inpatient service experts and collected suggestions from Veteran stakeholders exploring the relationship between the military service and deployment history and subsequent views of ACP of Veterans and their families. Several contributing themes emerge which are sorted in the CFIR framework including: the level of associated support services, formal inclusion of families, group identity, difference in the perceived outcome, time since military service, branch of service. This preparatory overview informs an ongoing QI project for increasing Veteran engagement in formal and informal ACP and SICC including formal and informal documentation.

